# Ultrasound-guided foam sclerotherapy vs. open surgical ligation for incompetent perforator veins: a retrospective cohort study

**DOI:** 10.3389/fsurg.2026.1808776

**Published:** 2026-05-29

**Authors:** Rongjiang Li, Yongke Luo, Pin Lv, Zhe Ji, Xiong Lu, Haiwei Chen

**Affiliations:** 1 Department of Ultrasound, Baoji People’s Hospital, Baoji, Shaanxi, China; 2 Department of General Surgery, Baoji People’s Hospital, Baoji, Shaanxi, China

**Keywords:** foam sclerotherapy, incompetent perforator veins, open ligation, ultrasound guidance, venous ulcer

## Abstract

**Purpose:**

To compare the efficacy and safety of ultrasound-guided foam sclerotherapy (UGFS) vs. open ligation for treating incompetent perforator veins (IPVs) and to identify independent risk factors for IPV recanalization following UGFS.

**Methods:**

Clinical data from 97 patients with IPVs treated at Baoji People’s Hospital between February 2019 and February 2022 were retrospectively analyzed. Patients were categorized into the UGFS group (Group A, *n* = 49) and the open surgery group (Group B, *n* = 48). All patients were followed up for at least 12 months. Perioperative parameters, clinical outcomes [perforator vein occlusion rates, Venous Clinical Severity Score (VCSS), ulcer healing], and postoperative complication rates were compared between groups. Generalized estimating equations (GEE) were employed to identify risk factors for recanalization in Group A, and Receiver operating characteristic (ROC) curves were generated to determine optimal cutoff values for these factors.

**Results:**

Group A had significantly shorter operative time (56.27 ± 9.70 vs. 68.60 ± 9.41 min, *P* < 0.05), less intraoperative blood loss (4.18 ± 2.37 vs. 8.85 ± 4.48 mL, *P* < 0.05), and lower 24-hour postoperative visual analogue scale (VAS) score [1 (2) vs. 3 (1), *P* < 0.05] than Group B, with no significant difference in postoperative hospital stay between the two groups (*P* = 0.517). Immediate occlusion rates were 100% in both groups. At 12 months, the vein-level occlusion rate was significantly higher in Group B (95.3%) than in Group A (86.8%) (OR = 3.060, 95% CI: 1.086–8.619, *P* = 0.034). However, no significant differences were found between groups regarding VCSS improvement or ulcer healing rates (*P* > 0.05). The total complication rate was lower in Group A (10.2%) than in Group B (22.9%), though this difference did not reach statistical significance (OR = 2.616, 95% CI: 0.833–8.213, *P* = 0.092). Multivariate GEE analysis identified perforator vein diameter >4.5 mm (OR = 5.501, 95% CI: 1.034–29.277, *P* = 0.046) and body mass index (BMI) > 27.2 kg/m^2^ (OR = 1.385, 95% CI: 1.067–1.798, *P* = 0.014) as independent risk factors for recanalization in Group A. ROC curve analysis confirmed the predictive utility of these thresholds.

**Conclusion:**

UGFS demonstrated a favorable safety and efficacy profile, supporting its role as a viable minimally invasive alternative. Treatment strategies should be individualized based on patient-specific characteristics, such as vein diameter and BMI.

## 1 Introduction

Chronic venous disease (CVD) is a prevalent vascular disorder globally, representing a significant public health burden. Its prevalence increases markedly with age, affecting approximately 10.4%–39.0% of the adult population worldwide (1, 2). CVD is characterized by venous hypertension, reflux, and structural abnormalities of the venous wall, presenting with a spectrum of clinical manifestations ranging from mild leg heaviness and pain to severe skin pigmentation, lipodermatosclerosis, and venous ulcers. This condition imposes substantial physical and psychological distress on patients and places a heavy economic strain on healthcare systems (1, 3, 4). Incompetent perforator veins (IPVs) play a pivotal role in the pathogenesis and progression of CVD (5). Consequently, precise treatment of IPVs has become a critical component of comprehensive CVD management.

Traditional open surgical ligation is effective but carries inherent drawbacks, including significant surgical trauma, high risk of incision infection, and prolonged recovery periods (6). Currently, the treatment paradigm for IPVs is shifting from open surgery towards minimally invasive techniques (7). Ultrasound-guided foam sclerotherapy (UGFS) has gained widespread clinical adoption due to its simplicity, cost-effectiveness, and favorable patient tolerance (8). This therapy induces venous wall fibrosis and occlusion through chemical destruction of endothelial cells (9), with ultrasound guidance enhancing injection precision. However, controversies persist regarding the efficacy and safety of UGFS for IPVs. Some studies suggest lower venous occlusion rates compared to thermal ablation or open surgery, and key factors influencing its efficacy remain incompletely elucidated (5, 10). Optimized individualized treatment strategies for patients with varying disease severities are yet to be established.

This study retrospectively analyzed clinical data from IPV patients treated at Baoji People's Hospital over a 3-year period. The clinical efficacy and safety of UGFS vs. traditional open surgical ligation were systematically compared, and independent risk factors for IPV recanalization following UGFS were further investigated, aiming to provide evidence-based recommendations for clinical decision-making.

## 2 Materials and methods

### 2.1 Subjects

This retrospective cohort study adhered to the STROBE guidelines. Consecutive patients diagnosed and treated for IPVs at Baoji People's Hospital between February 2019 and February 2022 were included. The study protocol was approved by the Institutional Review Board (No. 2025-018), and written informed consent was obtained from all patients. Inclusion Criteria: (1) Aged ≥18 years; (2) Preoperatively diagnosed with IPVs by ultrasound, defined as a perforator vein diameter >3.5 mm and reflux time >500 ms (11); (3) CEAP classification ranging from C4a to C6; (4) Unilateral lower extremity lesions; (5) Complete medical records. Exclusion Criteria: (1) Deep venous stenosis (>50%), thrombosis or moderate to severe reflux; (2) History of previous lower extremity venous surgery; (3) Complicated with lower extremity arterial diseases; (4) Coagulopathy; (5) Severe hepatic or renal insufficiency; (6) Mental illness or inability to cooperate with follow-up. Based on the treatment administered, patients were categorized into the UGFS group (Group A) or the open surgery group (Group B). The decision-making process relied on patient preference and clinical feasibility, precluding random allocation or strict adherence to preoperative anatomical screening protocols.

### 2.2 Methods

#### 2.2.1 Preoperative assessment of IPVs

Preoperative doppler ultrasound examinations were jointly performed by the lead surgeon and a dedicated sonographer. Patients were examined in a standing or sitting position. The diameter and reflux time of pathological perforator veins were measured and recorded.

#### 2.2.2 Treatment procedures

All procedures were performed by a single lead surgeon with over 10 years of experience in open and minimally invasive treatments for lower extremity superficial and perforator vein diseases.

##### 2.2.2.1 Ultrasound-guided foam sclerotherapy (Group A)

**Figure 1:**
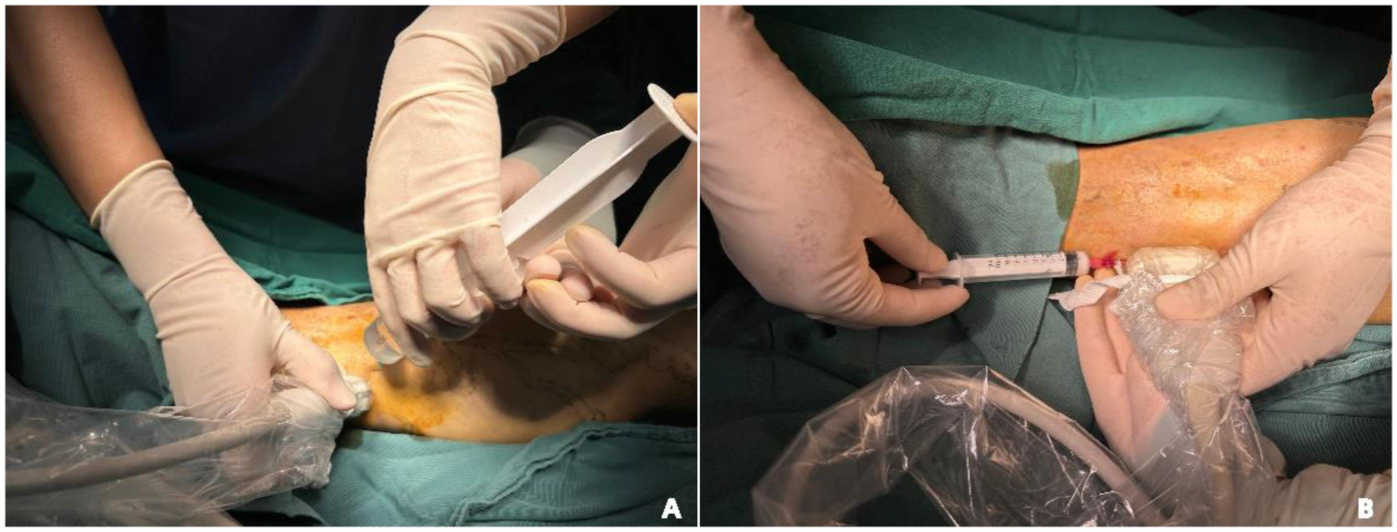
Intraoperative UGFS for IPVs. **(A)** Perivascular tumescent infiltration. **(B)** Foam sclerosant injection with proximal compression to prevent deep venous reflux.

**Figure 2:**
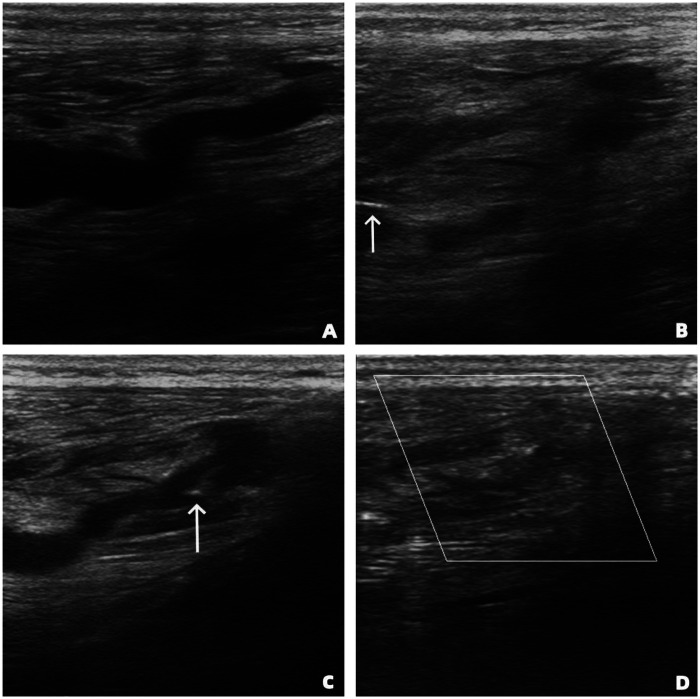
Intraoperative ultrasound imaging during UGFS. **(A)** Longitudinal view of the target vein. **(B)** Perivascular needle placement (arrow) and vein compression post-tumescent injection. **(C)** Intravenous foam injection (arrow) under real-time monitoring. **(D)** Post-procedure verification of flow occlusion.

1. Foam Preparation: Microfoam was prepared using the Tessari technique by mixing 1% polidocanol solution with air in a 1:4 ratio to achieve a uniform, fine consistency.
2. Tumescent Solution Preparation: The tumescent solution consisted of 250 mL 0.9% sodium chloride, 10 mL 2% lidocaine, and 5 mL 5% sodium bicarbonate.
3. Procedure: Under ultrasound guidance with the vein visualized in long axis, the prepared tumescent solution was injected percutaneously around the segment of the perforator vein adjacent to the deep vein to reduce its diameter, ideally achieving near-occlusion. A needle was then advanced into the lumen of the perforator vein beneath the deep fascia. Correct needle placement (confirmed by ultrasound showing the tip within the vein lumen) was verified. The surgeon manually compressed the proximal end of the perforator vein near the deep vein junction using fingers or the ultrasound probe to prevent foam reflux into the deep venous system. Under real-time ultrasound monitoring, 0.5–1.0 mL of foam sclerosant was slowly injected into each target perforator vein. Foam diffusion was monitored to ensure complete filling of the target vein. Following injection, the needle was withdrawn, and local compression was applied for 5 min with massage to evacuate blood. A repeat ultrasound examination confirmed complete occlusion (absence of flow signal) (Figures 1, 2).

##### 2.2.2.2 Open surgical ligation (Group B)

A 1–3 cm longitudinal incision was made at the preoperatively marked site. The deep fascia was incised, and the perforator vein was carefully dissected, ligated, and divided. Intraoperative ultrasound guidance was utilized for difficult cases.

##### 2.2.2.3 Anesthesia and concomitant superficial vein treatment

Two patients in Group A received local anesthesia; all other patients in Group A and all patients in Group B received combined spinal-epidural anesthesia. Both groups underwent concomitant treatment of superficial veins as indicated, including high ligation, phlebectomy, or foam sclerotherapy, alone or in combination.

##### 2.2.2.4 Postoperative management and follow-up

Postoperatively, limbs were wrapped with elastic bandages for 1 week and then used class II compression stockings full-time for 3 months. Patients with ulcers continued standard wound care. Follow-up visits were scheduled at 1, 3, 6, and 12 months postoperatively.

#### 2.2.3 Outcome measures

1. Safety Parameters: Perioperative metrics included operative time, intraoperative blood loss, 24-hour postoperative VAS pain score, postoperative hospital stay, and complication rates. Complications were graded according to the Clavien–Dindo classification (12).
2. Efficacy Parameters: Vein-level and patient-level perforator vein occlusion rates, VCSS, ulcer healing rate, and time to healing. Venous occlusion was defined as the absence of intraluminal flow on ultrasound; recanalization was defined as the presence of flow or reflux upon distal compression. Vein-level occlusion rates were calculated per individual perforator vein; patient-level occlusion rate was defined as the closure of all target perforator veins in a given patient.
3. Risk Factors for Recanalization and Cutoff Determination.

### 2.3 Statistical analysis

Statistical analyses were performed using SPSS version 26.0 and G-Power version 3.1.9.7. The Shapiro–Wilk test was used to assess the distribution of data. Normally distributed measurement data were expressed as mean ± standard deviation (*x¯*± *s*), while non-normally distributed measurement data were presented as median (interquartile range) [*M* (IQR)]. Count data were expressed as number (percentage) [*n* (%)]. Comparison between groups was conducted using independent samples *t*-test, Mann–Whitney *U* test, chi-square test or Fisher's exact test. Intragroup comparison was performed using paired *t*-test or Wilcoxon signed-rank test. *Post-hoc* power analysis for primary outcomes was performed using G-Power. Variables with *P* < 0.1 in univariate analysis were included in the multivariate model. GEE were used to analyze vein-level occlusion rates and risk factors for recanalization, correcting for intra-patient clustering effects. ROC curves were generated to determine optimal cut-off values. A two-sided *P*-value <0.05 was considered statistically significant.

## 3 Results

### 3.1 Baseline data

**Figure 3:**
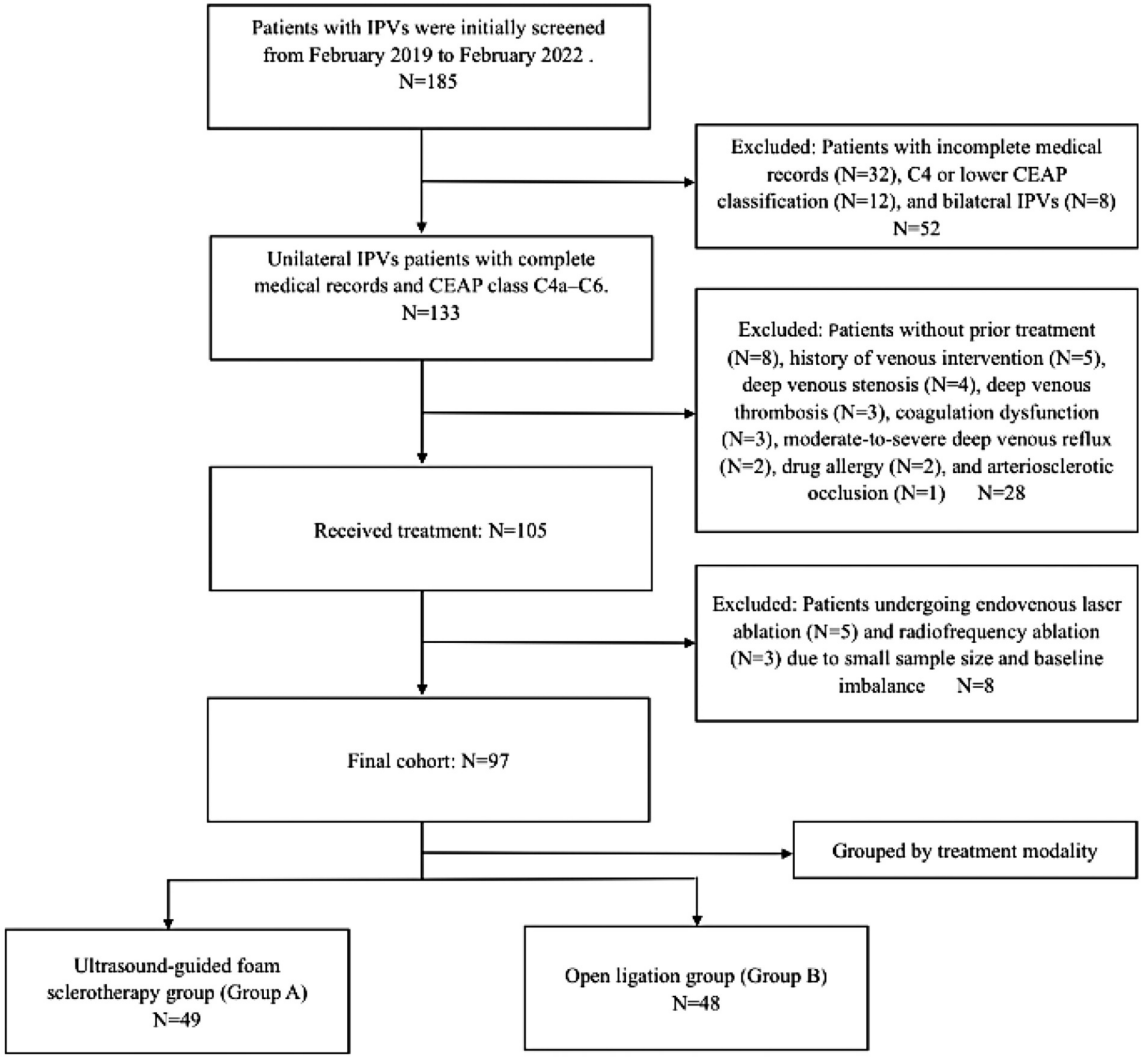
Patient screening flowchart.

Of 185 initially screened patients, 97 were ultimately included (Group A: *n* = 49; Group B: *n* = 48), comprising a total of 177 IPVs. The patient screening process is shown in Figure 3. Except for two patients in each group, all others underwent concomitant superficial vein surgery. The distribution of specific surgical procedures did not differ significantly between groups (*P* = 0.770). Baseline characteristics were well-balanced between the two groups (*P* > 0.05) (Table 1).

**Table 1:** Comparison of baseline characteristics between the two groups.

Item	Group A (*n* = 49)	Group B (*n* = 48)	*t*/*χ*^2^/*Z*	*P*-value
Gender [*n* (%)]			*χ*^2^ = 0.300	0.584
Male	29 (59.2)	31 (64.6)		
Female	20 (40.8)	17 (35.4)		
Age (years, *x¯* ± *s*)	58.45 ± 13.21	55.71 ± 12.27	*t* = 1.058	0.293
Disease duration [years, *M* (IQR)]	10 (8)	10 (11.5)	*Z* = −0.685	0.493
BMI (kg/m^2^, *x¯* ± *s*)	24.13 ± 3.19	23.46 ± 3.59	*t* = 0.976	0.331
CEAP classification [*n* (%)]			*χ*^2^ = 0.000	>0.999
C4a	25 (51.0)	21 (43.8)		
C4b	11 (22.4)	13 (27.1)		
C5	5 (10.2)	7 (14.6)		
C6	8 (16.3)	7 (14.6)		
Comorbidities [*n* (%)]			*χ*^2^ = 0.780	0.780
Hypertension	10 (20.4)	8 (16.7)		
Coronary heart disease	7 (14.3)	6 (12.5)		
Diabetes mellitus	3 (6.1)	4 (8.3)		
History of surgery [*n* (%)]	5 (10.2)	3 (6.3)	Fisher	0.715
IPVs per affected limb [*M* (IQR)]	2 (1)	2 (1)	*Z* = −0.501	0.616
Internal diameter [mm, *M* (IQR)]	4 (0.5)	3.8 (0.52)	*Z* = −0.092	0.927
Reflux time [ms, *M* (IQR)]	820 (376)	702 (411.25)	*Z* = −0.580	0.562

### 3.2 Safety parameters

Group A demonstrated significantly superior outcomes in operative time, intraoperative blood loss, and postoperative pain compared to Group B (*P* < 0.05). No significant difference was observed in postoperative hospital stay (*P* = 0.517). The total complication rate was lower in Group A (10.2%, 5/49) than in Group B (22.9%, 11/48); however, this difference did not reach statistical significance (OR = 2.616, 95% CI: 0.833–8.213, *P* = 0.092). Notably, the *post-hoc* power for this comparison was low (39.9%, effect size = 0.171), indicating a potential risk of Type II error. All complications were classified as Clavien–Dindo Grade II and resolved with symptomatic treatment; no serious adverse events occurred (Table 2).

**Table 2:** Comparison of perioperative indices between the two groups.

Item	Group A (*n* = 49)	Group B (*n* = 48)	*t*/*χ*^2^/*Z*	*P*-value
Operative time (min, *x¯* ± *s*)	56.27 ± 9.70	68.60 ± 9.41	*t* = −6.355	<0.001
Blood loss (mL, *x¯* ± *s*)	4.18 ± 2.37	8.85 ± 4.48	*t* = −6.403	<0.001
VAS [*M* (IQR)]	1 (2)	3 (1)	*Z* = −4.743	<0.001
Postoperative hospital stay (days, *x¯* ± *s*)	3 (2.5)	3 (2.75)	*Z* = −0.648	0.517
Complications [*n* (%)]			*χ*^2^ < 0.001	>0.999
Incision infection	0	3 (6.3)	Fisher	0.117
Thrombophlebitis	3 (6.1)	2 (4.2)		1.000
Deep venous thrombosis	0	0	–	–
Pulmonary embolism	0	0	–	–
Lower extremity numbness	2 (4.1)	6 (12.5)	Fisher	0.159
Subcutaneous hematoma	0	0	–	–
Total complications	5 (10.2)	11 (22.9)	*χ*^2^ = 2.845	0.092

### 3.3 Efficacy parameters

#### 3.3.1 Perforator vein occlusion rates

Immediate occlusion rates were 100% in both groups. At the patient level, the 12-month occlusion rate was numerically higher in Group B (91.7%) than in Group A (77.6%), but the difference was not statistically significant (OR = 3.184, 95% CI: 0.936–10.828, *P* = 0.090). The *post-hoc* power for this comparison was low (48.4%, effect size = 0.195), suggesting that a Type II error cannot be entirely ruled out. No significant differences were observed at other follow-up time points (*P* > 0.05) (Table 3). Further analysis using Generalized Estimating Equations (GEE) for vein-level data with repeated measures revealed that at 12 months, the vein-level occlusion rate was significantly higher in Group B than in Group A (OR = 3.060, 95% CI: 1.086–8.619, *P* = 0.034) (Table 4).

**Table 3:** Comparison of patient-level occlusion rates and VCSS scores between the two groups.

Item	Group A (*n* = 49)	Group B (*n* = 48)	*χ*^2^/*Z*	*P*-value
Occlusion rates [*n* (%)]				
1 month postop	48 (98.0)	48 (100.0)	–	>0.999
3 months postop	44 (89.8)	46 (95.8)	–	0.436
6 months postop	41 (83.7)	45 (93.8)	*χ*^2^ = 2.449	0.199
12 months postop	38 (77.6)	44 (91.7)	*χ*^2^ = 3.696	0.090
Preoperative VCSS score [*M* (IQR)]	9 (3)	10 (3.75)	*Z* = −0.614	0.539
Postoperative VCSS score [*M* (IQR)]				
1 month postop	6 (2.5)	7 (4)	*Z* = −1.070	0.285
3 months postop	5 (1)	6 (2)	*Z* = −0.929	0.353
6 months postop	4 (2)	4 (2)	*Z* = −0.939	0.348
12 months postop	3 (1)	4 (2)	*Z* = −1.072	0.284

**Table 4:** Comparison of vein-level occlusion rates between the two groups.

Item	Group A (*n* = 91)	Group B (*n* = 86)	*B*	*P*-value	OR (95% CI)
Occlusion rates [*n* (%)]					
1 month postop	90 (98.9)	86 (100.0)		>0.999	
3 months postop	85 (93.4)	84 (97.7)	1.064	0.119	2.897 (0.762–11.014)
6 months postop	82 (90.1)	83 (96.5)	1.024	0.057	2.783 (0.970–7.981)
12 months postop	79 (86.8)	82 (95.3)	1.118	0.034	3.060 (1.086–8.619)

#### 3.3.2 VCSS scores

VCSS scores in both groups decreased significantly at all postoperative follow-up points compared to baseline (*P* < 0.05). However, no significant differences were observed between the two groups at any time point (*P* > 0.05) (Table 3).

#### 3.3.3 Ulcer healing

Among 15 patients with CEAP C6 classification (8 in Group A, 7 in Group B), the ulcer healing rate was 100% in both groups. The mean time to healing was 34.13 ± 13.25 days in Group A and 37.00 ± 15.38 days in Group B, with no significant difference (*t* = 0.635, *P* = 0.703). No ulcer recurrences were observed during the 12-month follow-up.

### 3.4 Analysis of factors influencing perforator vein recanalization

Among the 91 perforator veins in Group A, 12 experienced recanalization. Univariate GEE analysis indicated that disease duration, BMI, vein diameter, and reflux time were associated with post-treatment recanalization (*P* < 0.1). Multivariate analysis identified perforator vein diameter (OR = 5.501, 95% CI: 1.034–29.277, *P* = 0.046) and BMI (OR = 1.385, 95% CI: 1.067–1.798, *P* = 0.014) as independent risk factors for recanalization (Table 5). ROC curve analysis demonstrated that a vein diameter >4.5 mm and a BMI >27.2 kg/m^2^ significantly increased the risk of recanalization (Table 6).

**Table 5:** GEE analysis of independent risk factors for IPV recanalization in Group A.

Variable	*B*	SE	Wald	*P*-value	OR (95% CI)
Internal diameter (mm)	1.705	0.853	3.995	0.046	5.501 (1.034–29.277)
BMI (kg/m^2^)	0.326	0.133	5.992	0.014	1.385 (1.067–1.798)

**Table 6:** ROC analysis of vein diameter and BMI cut-offs to predict recanalization in Group A.

Variable	Cut-off value	Youden's index	AUC	95% CI	*P*-value
Internal diameter (mm)	4.5	0.674	0.816	0.677–0.956	<0.001
BMI (kg/m^2^)	27.2	0.522	0.791	0.606–0.976	0.001

## 4 Discussion

Perforator veins (PVs) serve as crucial channels connecting the superficial and deep venous systems of the lower extremities. The normal lower limb contains approximately 90–150 PVs, with diameters ranging from 0.5 to 3 mm, predominantly located on the medial calf (13, 14). Incompetent perforator veins (IPVs) transmit high pressure to the superficial veins and capillary beds, leading to venous hypertension, tissue hypoxia and fibrosis, ultimately progressing to lipodermatosclerosis and venous ulcers (15). The ultrasound diagnostic criteria for IPVs are defined as a vein diameter >3.5 mm and a reflux time >500 ms (11). Such hemodynamic abnormalities have been confirmed as key predictors of CVD progression and venous ulcer recurrence (16, 17). Therefore, precise intervention targeting IPVs is a core component of comprehensive CVD management.

This retrospective study compared the efficacy of UGFS vs. open surgical ligation for IPVs. Both groups achieved 100% immediate success. However, UGFS demonstrated superior perioperative outcomes, including shorter operative time, less blood loss, and lower postoperative pain, consistent with its minimally invasive nature and the avoidance of fascial incisions (18, 19).

Reported short-term occlusion rates for UGFS range from 57% to 98% and decline gradually over time (19–21). In this study, patient-level occlusion rates were comparable between groups, whereas the 12-month vein-level occlusion rate was significantly lower in Group A (86.8%) than in Group B (95.3%). GEE and ROC analyses identified identified vein diameter >4.5 mm and BMI >27.2 kg/m^2^ as independent predictors of recanalization after UGFS. Mechanistically, larger-caliber veins have thicker walls that limit full foam penetration, resulting in incomplete endothelial injury and subsequent recanalization (22, 23). High shear stress also destabilizes foam structure, reducing treatment efficacy. In obese patients, excess subcutaneous fat impairs ultrasound visualization and increases venous hypertension, both of which promote recanalization. These findings are supported by studies showing that vein diameter >6 mm and severe obesity are linked to lower UGFS success rates (24–26). Hager et al. (19) further confirmed that BMI >50 kg/m^2^ predicted treatment failure across all perforator vein therapies, with an occlusion rate of only 37%. Univariate analysis indicated that disease duration and reflux time were associated with recanalization (*P* < 0.1), but these were not independent predictors in the multivariate model. Foam characteristics, injection technique, vasospasm, and postoperative compression may also affect UGFS outcomes (27, 28).

Despite the lower anatomical occlusion rate in Group A, clinical improvement and ulcer healing were equivalent between groups. At 12 months, VCSS scores were significantly and comparably reduced in both groups. Among 15 CEAP C6 patients, ulcer healing rates were 100% in both groups, with similar healing times and no recurrence. These results indicate that recanalization does not equate to clinical failure (29). In Group A, recanalized vessels showed reduced diameter and reflux and no longer met IPV diagnostic criteria, suggesting attenuated hemodynamic severity that does not drive disease progression (30). Concomitant superficial vein treatment further relieved venous hypertension, mitigating the impact of perforator recanalization (31).

The overall complication rate was lower in Group A (10.2%) than in Group B (22.9%), though not statistically significant, confirming the safety of minimally invasive treatment. Group B had three cases of incision infection, consistent with the high infection risk in tissues with trophic changes (6). UGFS uses percutaneous puncture without incisions, eliminating wound-related complications, in line with Ho et al. (6). Superficial thrombophlebitis was mild and comparable between groups, attributable to sclerosant irritation and venous stasis (32); postoperative compression and early ambulation reduced this complication. The rate of lower extremity numbness was higher in Group B (12.5%) than in Group A (4.1%) due to nerve traction during open dissection (33), as cutaneous nerves near the medial calf closely accompany perforator veins (34). UGFS avoids extensive dissection and markedly reduces nerve injury risk (7, 18). All numbness resolved within 3–6 months without permanent deficits. No DVT or PE occurred in either group. In Group A, ultrasound guidance, tumescent infusion, proximal compression, and early mobilization prevented foam reflux into deep veins, yielding a DVT rate <1% as reported in the literature (33). Group B had no DVT because deep venous structures were not manipulated.

Minimally invasive alternatives for IPVs also include radiofrequency ablation (RFA) and endovenous laser ablation (EVLA). Several studies (7, 35) indicate that RFA and EVLA achieve higher long-term occlusion rates than UGFS. These thermal modalities induce venous wall fibrosis via heat, resulting in more thorough occlusion, particularly suitable for larger-diameter perforator veins (35). However, RFA and EVLA carry risks such as difficulty in cannulation and thermal injury, along with higher costs and lower patient tolerance compared to UGFS (36). Compared to RFA and EVLA, UGFS offers advantages of operational simplicity, lower cost, and the ability to treat multiple perforator veins and surrounding varicose tributaries simultaneously (19). For patients with multiple small incompetent perforator veins, UGFS can occlude multiple vessels via a single puncture and injection, whereas RFA and EVLA require sequential cannulation, making the procedure relatively more complex (37).

This study has several limitations. First, as a single-center retrospective cohort study with a non-randomized design, it is susceptible to selection bias and unmeasured confounding factors. Second, since all patients underwent concomitant superficial vein procedures, the specific contribution of perforator vein treatment to symptom improvement could not be independently assessed. Furthermore, the relatively small sample size resulted in low statistical power (<50%) for certain comparisons, necessitating cautious interpretation of negative findings due to the risk of Type II errors. The 12-month follow-up period is insufficient to evaluate long-term recurrence rates and health economic benefits, and key operational parameters, such as foam dosage, were not quantitatively recorded. Future multi-center, large-scale randomized controlled trials with standardized protocols and extended follow-up are urgently needed to further validate the independent efficacy of UGFS.

UGFS for the treatment of IPVs offers significant advantages, including shorter operative time, minimal trauma, and reduced postoperative pain. While its 12-month vein-level occlusion rate is lower than that of open surgical ligation, UGFS demonstrates comparable efficacy in alleviating clinical symptoms (VCSS) and promoting venous ulcer healing, with a favorable safety profile. Its operational simplicity and good patient tolerance make it an effective minimally invasive option for IPVs. In clinical practice, treatment strategies should be individualized based on patient-specific characteristics such as perforator vein diameter, BMI, and patient preference.

## Data Availability

The original contributions presented in the study are included in the article/Supplementary Material, further inquiries can be directed to the corresponding author.
